# The Mycetoma Research Center, University of Khartoum, Sudan’s experience in community engagement initiatives spans 3 decades

**DOI:** 10.1371/journal.pntd.0012304

**Published:** 2024-08-22

**Authors:** Ahmed Hassan Fahal, Eiman Siddig Ahmed, Ahmed Hussein Mahmoud, Ali Awadella Saaed

**Affiliations:** Mycetoma Research Center, Soba University Hospital, University of Khartoum, Khartoum, Sudan; Albert Einstein College of Medicine, UNITED STATES OF AMERICA

## Abstract

Mycetoma profoundly affects marginalised communities, especially in impoverished and remote areas with limited access to healthcare. This chronic and debilitating inflammatory disease highlights the typical issues of neglected tropical diseases (NTDs), such as insufficient attention, funding, and resources, which perpetuate neglect and suffering. Patients often delay seeking medical help, leading to advanced disease stages, severe complications, and lasting disabilities. The lack of medical infrastructure and skilled healthcare professionals worsens the situation, causing delays in diagnosis and inadequate treatment. Engaging affected communities in tailored interventions is essential to tackle these challenges, promote collaboration, raise awareness, and mobilise resources to improve healthcare access and enhance diagnostic and treatment capabilities. Since 1991, the Mycetoma Research Center (MRC) at the University of Khartoum, Sudan, has led community engagement initiatives aimed at improving the quality of life for mycetoma-affected individuals through education, advocacy, and local collaboration. In this communication, the MRC shares its extensive experience in community engagement to benefit mycetoma-affected communities.

## Background

Designated as a neglected tropical disease (NTD), mycetoma exemplifies key features of NTDs, such as a disproportionate impact on marginalised communities and inadequate attention and resources allocated to its control and management [1]. This designation underscores insufficient funding, research, and public health efforts compared to other diseases, thereby perpetuating a cycle of neglect and suffering within affected communities [2,3]. Mycetoma, a chronic, destructive, and disabling inflammatory disease, places a heavy burden on individuals, families, communities, and the health system, notably in impoverished, isolated regions with limited healthcare accessibility [4]. It predominantly impacts the most economically disadvantaged sectors of society, where essential healthcare, education, and resources are lacking [5,6]. Consequently, those affected often delay seeking medical assistance, leading to presentation at an advanced disease stage, which further carries the risk of serious complications [7,8]. This delay worsens the already considerable challenges in effectively managing mycetoma, increasing the likelihood of enduring incapacities, disabilities, and disfigurement [9].

In remote communities where mycetoma occurs, a lack of medical infrastructure exacerbates the situation. Access to skilled healthcare professionals, diagnostic tools, and essential medications is often severely restricted and may also be unaffordable [10]. This scarcity of resources results in involuntary healthcare rationing, leading to delays in diagnosis, inadequate treatment, and unfavourable health outcomes for individuals grappling with mycetoma [11]. Moreover, even in resourced clinical settings, the existing diagnostic and therapeutic tools for the management of mycetoma are inadequate, exacerbating the burden of the disease. The available diagnostic techniques frequently lack accuracy and accessibility, leading to delays in initiating appropriate treatment [12,13]. Additionally, available treatment options may be limited in effectiveness or carry significant side effects as well as costs, further complicating the management of mycetoma [14–17].

Engagement with communities is essential in addressing the challenges posed by mycetoma and reducing its impact on society. Involving affected local communities, media, local leaders, and healthcare providers allows tailored interventions to address specific needs and obstacles related to the disease [18]. This involvement fosters collaboration, raises awareness, and mobilises resources to improve healthcare access, enhance diagnostic capabilities, establish effective treatment strategies, and promote local community hygiene and environment. Empowering mycetoma-affected communities enables individuals to advocate actively for their health and well-being. Initiatives such as health education campaigns, community environment enhancement, empowerment of community health workers, and collaboration with local entities enable communities to advocate for better healthcare infrastructure, increased access to essential medications, and advancements in diagnostic and therapeutic approaches, as well as community improvement strategies for mycetoma [19].

The challenges posed by mycetoma are significant, particularly in resource-limited settings. Addressing these challenges necessitates a comprehensive approach emphasising community engagement, advocacy, and collaborative efforts to enhance healthcare access, raise awareness, and develop effective interventions to alleviate the burden of the disease [10]. Through this communication, the Mycetoma Research Center, WHO Collaborating Center on Mycetoma and Skin NTDs, is sharing the insights and lessons gained from its extensive experience in community engagement spanning 3 decades.

Since its inception in 1991, the Mycetoma Research Center (MRC), University of Khartoum, Sudan, has actively participated in numerous pioneering community engagement initiatives aimed at enhancing the quality of life for affected patients and communities. Here are some examples of these initiatives [20].

### Awareness campaigns

In 1994, the MRC launched its first-ever community survey and awareness campaign in partnership with the local authorities of Abu Gota Locality in El Gezira State, a highly endemic region, along with the enthusiastic participation of medical students from the University of Khartoum. Following this successful inaugural event, the MRC extended its efforts by organising multiple awareness campaigns across various mycetoma endemic regions of the country. Notably, a significant number of these campaigns were held in Sennar State, where both the local government and communities provided substantial support and provision for these initiatives [18].

These campaigns formed integral parts of the Mycetoma Research Center’s community engagement strategies, addressing mycetoma as a complex medical, health, and socioeconomic challenge and a debilitating illness. Their primary aim is to bridge the knowledge gap surrounding mycetoma among community members and to enable early case detection and management. Many individuals in affected regions may lack awareness of this disease due to limited access to healthcare services, insufficient health education, or cultural beliefs. Therefore, raising awareness through targeted campaigns is imperative to ensure early detection, prompt treatment, and prevention of further consequences.

A variety of channels were employed to conduct these awareness campaigns, catering to the diverse needs and preferences of the different communities. These included local radio, television, newspapers, and mobile audiovisual units visiting different villages. Local radio broadcasts, particularly in rural areas, reach a wide audience where access to other media forms may be limited. Radio and television programmes featuring discussions, interviews with healthcare professionals, and informational segments effectively conveyed key messages about mycetoma and promoted health-seeking behaviour [10].

Moreover, posters, booklets, and flyers were disseminated in community centres, healthcare facilities, schools, and other public spaces frequently visited by the MRC team and community members. These visual aids contained simple and informative content about mycetoma, including its presentation, modes of transmission, and the importance of early diagnosis and treatment.

Community meetings and gatherings provided valuable opportunities to engage directly with residents and offer in-depth information about mycetoma. These sessions took place at mosques after Friday prayers, schools, community clubs, and through house-to-house visits. Through interactive presentations and Q&A sessions, the MRC teams trained healthcare professionals, school teachers, and community leaders to address misconceptions and dispel myths about mycetoma. Additionally, mobile audiovisual units showing awareness videos moved between villages, encouraging individuals to seek medical assistance if they suspect they have mycetoma, were utilised.

In the digital era, social media platforms serve as powerful tools for reaching diverse audiences, including younger generations and urban populations. The MRC’s community engagement activities utilised these platforms to share educational content, raise awareness about mycetoma, and facilitate online discussions and support groups for affected individuals and their families.

Local health and administrative authorities, along with the MRC and numerous NGOs, provided financial backing for these initiatives. They have proven cost-effective, as a significant number of patients have begun seeking care at an early stage, leading to favourable treatment outcomes.

### Training and capacity building

Training and capacity-building initiatives are integral components of community engagement efforts aimed at effectively addressing mycetoma and its consequences. These initiatives were designed to educate and empower various stakeholders, including local healthcare providers, school teachers, community leaders, and Red Crescent volunteers, to recognise, manage, refer, and respond to the challenges presented by mycetoma within their communities.

At Sennar State, a highly mycetoma-endemic area, all medical assistants were trained in mycetoma awareness and referrals, while medical assistants, nurses, and health workers in the Eastern Sennar Locality, a highly endemic locality received more in-depth training in mycetoma presentation and ultrasound examination at the Wad Onsa Mycetoma Satellite Center. These training programmes equipped them with the skills to conduct thorough assessments, make timely diagnoses, and avoid inappropriate practices, such as performing surgery under local anaesthesia in unsuitable settings. All these activities were performed under the patronage of the State Governor and Minister of Health to emphasise their importance.

Community leaders and activists also underwent training sessions to deepen their understanding of mycetoma’s impact on public health, the significance of early detection and treatment, and the improvement of the local community’s hygiene and environment and to reduce the risk of stigmatising affected individuals. Armed with this knowledge, they advocated for improved healthcare access, promoted health-seeking behaviours, improved villages’ environment and hygiene, and facilitated collaboration between local stakeholders and healthcare providers.

Moreover, local Red Crescent volunteers played diverse roles in mycetoma awareness and management, conducting targeted outreach activities, facilitating health screening, providing educational workshops, and acting as intermediaries for timely referrals, collectively contributing to enhanced health outcomes for mycetoma-affected individuals within communities.

### Engaging volunteers

Engaging volunteers is crucial in community-based healthcare and socioeconomic support initiatives, especially in areas with limited resources, where they act as crucial communicators between healthcare services providers and underserved populations, notably in tackling health challenges such as mycetoma. The MRC team trained numerous community volunteers to identify mycetoma symptoms for early detection and management, empowering them to become influential figures within their communities. These trained volunteers carried out several targeted outreach activities, including health screenings, offered basic education on prevention and management, and assisted in referring suspected cases and environmental samples collections for research. Additionally, the team provided a volunteer vehicle for transporting patients and medications, all contributing to heightened awareness, early detection, and enhanced management of mycetoma, thereby significantly improving the lives of affected individuals. One of these volunteers received recognition as a Mycetoma Ambassador, acknowledging their exceptional contributions and accomplishments.

### Disseminating precise information

Disseminating accurate information is not only vital but also fundamental in the efforts to raise awareness and educate communities about mycetoma, thereby dismissing myths and empowering individuals to take proactive steps in addressing the disease and its consequences. To achieve this, the MRC teams have undertaken numerous training sessions across various mycetoma-endemic regions, targeting a diverse range of stakeholders, including local universities and higher medical and health training centres staff and students, schools teachers and pupils, and local political and administrative authorities. Notably, meetings, presentations, and focus group discussions have been organised under the auspices of esteemed figures such as the State Governors and Ministers, demonstrating a high-level commitment to addressing mycetoma. These training programmes were meticulously designed to equip participants with precise and up-to-date knowledge about mycetoma, covering a wide array of aspects, including its aetiology, transmission, and management. Through these sessions, attendees acquire a comprehensive understanding of the disease, its implications, and the importance of early detection and intervention. Moreover, preventive measures are emphasised, advocating for simple yet crucial hygiene practices and the prompt seeking of medical attention for suspicious lesions. To enhance the effectiveness of these training initiatives, a variety of educational materials have been developed, including visually engaging posters, informative booklets, educational videos, impactful audio messages, and even catchy songs, ensuring that information is conveyed in a manner that resonates with diverse audiences. Local traditional healers play a crucial role in patient care and livelihoods in numerous endemic areas. Our plan is to educate and persuade them to encourage early case detection and referral for patients [14].

### Community-based surveillance

The establishment of community-based surveillance systems is crucial for early detection, management, and reporting of mycetoma cases, presenting a proactive approach to monitoring and tracking the disease’s prevalence in communities. The MRC has laid out a framework for such surveillance. A survey team comprising members from the MRC, medical students from the University of Khartoum and the University of Sennar, health staff from the State of Sennar, and community activists has been established. Employing cluster sampling, the MRC surveyed the Eastern Sennar locality, selecting 60 villages randomly across the locality’s 5 administrative units and conducting household-to-household surveys. A total of 41,176 individuals were surveyed, resulting in the identification of 359 suspected mycetoma patients. Suspected patients were referred to the Wad Onsa Mycetoma Satellite Centre for diagnosis and treatment. This surveillance facilitated the gathering of valuable epidemiological and clinical data on mycetoma. The overall prevalence of mycetoma was found to be 0.87% (95% CI = 0.78% to 0.97%), with a prevalence of 0.83% (95% CI = 0.71% to 0.96%) among males and 0.92% (95% CI = 0.79% to 1.06%) among females. The age group 31 to 45 years exhibited the highest prevalence among different age groups (1.52%, 95% CI = 1.23% to 1.86%) [21–25].

A prevalence map indicated the clustering of patients in the central and northeastern parts of the locality, with few or no cases in villages in the southwestern part, was generated. The collected data will aid in designing measures to alleviate the disease burden in the state, and the survey procedures and protocols can be replicated for further studies in Sudan and beyond and help to identify areas of higher risk [21–25].

Moreover, these surveys enhanced the team’s capabilities in identifying, reporting, and responding to suspected mycetoma cases. The team received training programmes that encompassed disease recognition, data collection techniques, communication strategies, and referral protocols, equipping team members with the requisite knowledge and skills to contribute effectively to surveillance efforts. Such survey initiatives offer avenues for interdisciplinary collaboration, fostering mutual understanding, shared objectives, and coordinated endeavours to combat mycetoma within the community.

### Stigma reduction

Mycetoma is a disabling, stigmatising medical and socioeconomic disease [9,26]. Efforts to reduce the stigma surrounding mycetoma are crucial to addressing the social, economic, and psychological hurdles faced by affected individuals and families, often due to visible deformities, disabilities, and secondary bacterial infection. Stigma arises from misconceptions and discrimination, leading to social and community isolation and barriers to healthcare. Our community engagement activities have been vital in combating this stigma through health education, advocacy, and empathy-building. Initiatives focused on dispelling myths, empowering affected individuals and communities, and fostering understanding through open dialogue and collaborative partnerships were conducted.

The MRC orchestrated diverse community engagement endeavours aimed at nurturing empathy and understanding towards individuals afflicted with mycetoma. These initiatives encouraged open discourse, compassion, and mutual regard, incorporating various elements such as storytelling by affected individuals and families, testimonies, and interactive sessions. Their purpose was to cultivate empathy among community members, healthcare providers, and policymakers. Through these engagements, the human repercussions of mycetoma were underscored, emphasising a shared sense of humanity. Consequently, a culture of acceptance and support was fostered, leading to diminished stigma and discrimination. Numerous documentaries addressing these issues have been created and made accessible online [20]. Notably, many of these activities featured prominent Sudanese figures such as celebrities, comedians, journalists, and programme producers who had been educated on the multifaceted aspects of mycetoma to promote a vision of hope.

### Outreach villages-based clinical services

Mycetoma, a neglected tropical disease, poses severe health risks if left untreated, often leading to irreversible damage and disabilities [8,9,27]. Due to socioeconomic challenges, affected individuals from underdeveloped communities typically seek medical help at advanced stages of the disease. To address these issues, 2 mycetoma regional satellite centres, in highly mycetoma-endemic regions in Sudan were established. They offer comprehensive care, including disease diagnosis, surgical treatment, health education, and prostheses, free of charge. These centres also aim to enhance early case detection and management, conduct research, provide socioeconomic support, and raise awareness about mycetoma [10,28].

The satellite centres are equipped with a range of essential facilities aimed at facilitating comprehensive care for mycetoma patients. These include state-of-the-art surgical complex units, patient wards furnished with necessary amenities, out-patient units dedicated to regular check-ups and appointments, advanced ultrasound suites capable of high-quality imaging, fully equipped laboratories specialising in microbiological and molecular testing, telemedicine units facilitating seamless communication with the MRC, and pharmacies offering free medication storage and dispensation.

These well-equipped facilities play a critical role in ensuring prompt diagnosis, treatment, and management of mycetoma cases within the region. By integrating various specialised services under one roof, the satellite centres strive to provide holistic care and support to patients affected by this NTD [10,28].

Furthermore, the satellite centres have served as vital hubs for healthcare outreach and community engagement initiatives. Over the years, they have hosted numerous MRC activities and events aimed at serving the local population. Thousands of suspected patients have been examined, with over 1,000 undergoing surgical treatment. Additionally, the centres have conducted approximately 2,000 free ultrasound examinations, organised various health education events, conducted field surveys to gather epidemiological data, and hosted several socioeconomic support events. Through these multifaceted endeavours, the satellite centres have not only contributed significantly to the diagnosis and treatment of mycetoma but also have played a crucial role in raising awareness, promoting education, and addressing the socioeconomic challenges faced by affected individuals within the community.

### Awareness and advocating for mycetoma in endemic villages

In collaboration with local health authorities in regions prone to mycetoma, the MRC has spearheaded a variety of awareness and advocacy campaigns. These initiatives have involved collaboration with prominent Sudanese conceptual visual artists, musicians, and photographers. They have conducted numerous health education sessions across diverse endemic communities, schools, and mycetoma satellite centres, focusing on the disease’s presentation, treatment, and control. Moreover, the team has organised multiple open-air theatre performances in numerous villages, capturing countless photographs and videos, and curating a photo exhibition to amplify their mycetoma advocacy efforts and raise public awareness.

Health Promotion Teams from various national and international associations and societies, alongside local community youth organisations, have arranged a range of health awareness events. These efforts employ diverse methods and health education materials, including the distribution of mycetoma-themed notebooks to students and children, as well as the dissemination of leaflets and posters. Members of the local community youth organisations actively engage in various performances and interactive theatre shows to promote awareness and advocacy. Moreover, the local Red Crescent Organisations have also conducted numerous advocacy sessions, including door-to-door outreach programmes [10].

Local radio and television stations broadcast numerous interviews and programmes, extensively publicising the missions’ activities and encouraging individuals to seek free treatment prior to the MRC missions arrival in villages. To facilitate this, local medical assistants and community leaders have been mobilised to direct suspected patients to the Wad Onsa Mycetoma Satellite Centre for diagnosis confirmation and treatment.

### Mycetoma ambassadors

Over the years, numerous distinguished and renowned individuals from various backgrounds have dedicated themselves to supporting mycetoma patients, aiming to enhance their quality of life. These individuals have been recognised and honoured as Mycetoma Ambassadors. These illustrious individuals have exhibited exemplary commitment, generously contributing their time, energy, and passion to raise understanding and awareness about mycetoma. Their steadfast support has been critical in nurturing a sense of community and shedding light on the challenges faced by those affected by mycetoma. We take great pride in acknowledging and celebrating these Mycetoma Ambassadors for their exceptional accomplishments in positively impacting the lives of individuals affected by this condition.

### Utilising music, drama, and fine art: A novel method for enhancing mycetoma health awareness and advocacy

Late presentation of mycetoma leads to significant negative physical and socioeconomic impacts and stigma on patients, their families, and communities in endemic regions [10,29]. Early detection and proper management necessitate comprehensive health education and awareness among patients. In pursuit of this goal, the Mycetoma Research Center has appointed Mr. Asim EL Bana, a renowned celebrity in Sudan, as the Mycetoma Awareness and Advocacy Ambassador. He spearheaded the establishment of a mycetoma awareness and advocacy group called **“We Are With You,”** comprising over 40 prominent Sudanese celebrities, including singers, musicians, journalists, poets, actors, radio and TV personalities, and reporters. The group’s mission is to raise awareness and provide health education for the early detection and management of mycetoma. They have engaged in various activities, including media interviews on television, radio, and in the press, as well as active participation on social media platforms [30].

On August 7, 2017, the **“We Are With You”** group held its inaugural event at the MRC. The event included poetry recitals and musical performances, engaging patients and their companions. Patients expressed gratitude and experienced a sense of joy and unity in the waiting area at the centre.

Subsequently, the group organised a campaign and festival for mycetoma advocacy in Sennar State. The campaign, held on October 10, 2017, commenced in Khartoum and included stops in several major cities en route to Sennar. During the campaign journey, MRC staff provided education on mycetoma, emphasising key facts for effective communication during the campaign. The campaign received support from government officials in Sennar and AL Gazeria States, with senior officials accompanying the campaign and endorsing its efforts at various stops along the route.

Each city welcomed the campaign, acknowledging the vital role of music, poetry, art, and media in mycetoma advocacy and health education. Campaign members addressed the crowds, emphasising disease prevention, early presentation for diagnosis and treatment, and the importance of protective measures during agricultural activities.

The campaign culminated in Wad Madani, where AL Gazeria Television hosted an interview with campaign members and the State Minister of Culture and Information, highlighting the role of Sudanese artists in health education, particularly regarding mycetoma.

In Sennar State, the campaign received a warm welcome from the State Governor and community leaders, with traditional dance performances adding to the celebratory atmosphere. The campaign concluded at the City Cultural Centre with speeches, performances, and a strong message urging the audience to prioritise mycetoma prevention and early intervention. The use of music, fine art, and drama in mycetoma advocacy marked a pioneering effort in Sudan, yielding positive impacts on awareness and advocacy for the disease.

A 27-min film addressing mycetoma awareness, advocating for early presentation and management, aiming to reduce stigma, especially surrounding mycetoma limb amputation, was created. Notably, this film constitutes a component of a Master’s degree in art pursued by a staff member of the MRC, filmed in a mycetoma-endemic village.

### The mycetoma visual documentation workshop

This training workshop was held on March 13th, 2022 and marked a significant milestone in the intersection of fine and applied art and medical sciences. Esteemed dignitaries, including the Minister of Higher Education and Scientific Research and the Vice-Chancellor of the Sudan University of Science and Technology, graced the event with their presence. This gathering drew a diverse audience comprising professors, students, and various interest groups, all convened with a shared objective to bridge the realms of fine and applied arts with medical sciences in order to amplify awareness surrounding diseases such as mycetoma.

Throughout the workshop, speakers underscored the imperative of amalgamating fine arts and social sciences with medical sciences to communicate health-related information effectively. They lauded the workshop’s essential role in enhancing societal awareness and emphasised the transformative potential of visual arts, music, and drama in fostering dialogue and disseminating crucial information on mycetoma. Furthermore, they applauded the collaborative efforts between educational institutions and the artistic community, recognising their collective endeavour in serving society. Moreover, they reiterated the Fine and Applied Art College’s steadfast commitment to heritage preservation and the promotion of societal values.

Spanning 7 days, the workshop witnessed the active participation of 13 fine and applied artists and their students. Over the course of the event, more than 70 ceramic collections were meticulously crafted, each serving as a tangible testament to the fusion of artistic expression with mycetoma awareness. The practical sessions on ceramic making provided hands-on learning experiences, complemented by educational films and an integrated exhibition aimed at illuminating the community about the complexities of mycetoma and its far-reaching consequences.

### Media professionals training on mycetoma awareness

The MRC, in partnership with the Hope International Organisation, coordinated a comprehensive three-day training workshop geared towards fostering mycetoma health promotion, advocacy, and awareness among media professionals. This transformative event unfolded between the 2nd and 4th of January 2021, drawing together a diverse cohort of 20 participants representing various media specialities. Among them were comedians, journalists, radio and TV commentators, newspaper writers, poets, actors, fine artists, film editors and producers, public relations specialists, bloggers, sound engineers, and art directors [20].

Structured to provide a multifaceted learning experience, the training encompassed a range of activities, including presentations, video showcases, small group discussions, interactions with patients, and tours of different MRC departments and units. Participants were exposed to invaluable insights into mycetoma, delving into its psychological, medical, health, and socioeconomic implications on individuals, families, and communities. Additionally, the programme delved into the strategic utilisation of social media technology, photography, comedy, and the press in mycetoma advocacy and awareness campaigns, emphasising the need to craft objective videos and films.

Miss Lymia Mutmakal, a revered Senior Sudan Radio commentator and programme producer, delivered a poignant presentation sharing her personal journey as a cancer survivor. Her narrative underscored the critical role of the media in offering support to individuals grappling with health challenges like mycetoma.

The participants echoed the significance of leveraging fine art and music to address the profound impact of mycetoma on patients and communities. They stressed the importance of health education in facilitating early detection and management of the condition.

The workshop fostered meaningful interactions between participants and mycetoma patients, providing firsthand insights into the disease’s socioeconomic implications. Notably, a former singer-turned-patient shared his harrowing experience with mycetoma, offering a touching rendition of his journey through song. His presence and contribution lent a humanising dimension to the discussions.

Following rigorous brainstorming and group deliberations, participants collaboratively devised a roadmap for the Mycetoma Media Advocacy Programme. Each group presented their proposals, complete with practical demonstrations, showcasing the innovative approaches they envisaged for raising awareness about mycetoma.

In a reflective closing ceremony, participants expressed their gratitude for the training workshop and affirmed their unwavering commitment to supporting mycetoma patients and the MRC. They pledged to forge close and active collaborations aimed at extending support to patients and their families, thus underscoring the enduring impact of the workshop on fostering solidarity within the media community for the cause of mycetoma awareness and advocacy [20].

### The mycetoma cultural week

A collaborative initiative between the Mycetoma Research Center, the French Institute, and the French Embassy in Khartoum took place from February 23rd to 27th, 2020, hosted at The French Institute in Khartoum, Sudan, with the esteemed Patronage of HE, The French Ambassador to Sudan.

With the absence of any established prevention or control program for mycetoma, early case detection and treatment remain paramount in alleviating the burden of this debilitating condition [1,4,5,7]. In response, the MRC orchestrated the Mycetoma Cultural Week, a multifaceted event aimed at harnessing the fusion of science and art. Its objectives encompassed enhancing public awareness, diminishing the stigma surrounding mycetoma, engaging stakeholders in advocacy endeavours, and leveraging fine arts as a medium for societal impact.

The Cultural Week commenced with an opening ceremony graced by notable figures, including the Vice Chancellor of the University of Khartoum, the Minister of Higher Education and Scientific Research, and the French Ambassador to Sudan. These dignitaries lauded the MRC’s commendable achievements in combating mycetoma, commending the center’s innovative approach in organising such a culturally enriching event.

A highlight of the Cultural Week was the Fine Art Exhibition, showcasing an impressive collection of artworks generously contributed by 14 renowned artists. Complemented by compelling videos addressing mycetoma advocacy and awareness, the portrayal exhibition catalysed intensified advocacy efforts and garnered widespread empathy for mycetoma patients.

Attendees were further enriched by enlightening lectures, presentations, and small group discussions that shed light on the holistic management of mycetoma patients delivered during the Cultural Week. The comprehensive discourse delved into various facets of mycetoma, with a particular emphasis on its social impact and the MRC’s concerted efforts to alleviate its burdens.

The Cultural Week was crowned by a prestigious closing ceremony attended by the Director of the French Institute, who commended the artists for their outstanding contributions. Expressing gratitude to all collaborators, the Director underscored the event’s success in fostering enlightenment, advocacy, and solidarity in the ongoing fight against mycetoma. Attendees were duly honoured with awards and certificates, marking the zenith of a week dedicated to raising awareness and advancing the cause of mycetoma advocacy.

### The community environment improvement campaigns

These campaigns initiated in Wad El Nimear village, a highly endemic mycetoma area in Sudan with extremely poor hygiene, exemplify a concerted effort to address the root causes of mycetoma transmission and improve overall public health. Led by the Mycetoma Research Center in collaboration with the Sennar State Government, local authorities, community leaders, activists, and Red Crescent volunteers, these campaigns aimed to create a safer and more hygienic environment for the villagers [10].

One crucial aspect of these campaigns was the removal and disposal of thorny trees, bushes, and animal enclosures. These efforts targeted the reduction of mycetoma transmission risk factors, such as puncture wounds from thorns and sharp objects and exposure to animal dung, which are known contributors to the spread of the disease. By eliminating these hazards, the community members took a proactive step towards mitigating the incidence of mycetoma infections.

Furthermore, the establishment of 72 contemporary animal enclosures beyond the village confines marks a substantial stride toward bolstering hygiene practices and diminishing close interaction between villagers and animals. This endeavour not only mitigates the potential for mycetoma transmission but also enhances public health on a broader scale by curbing the dissemination of zoonotic illnesses and elevating sanitation norms. This endeavour, funded by a philanthropic donation from an engineering firm, underscores the significance of corporate social responsibility in community advancement. Their support not only facilitated the construction of these modern enclosures but also served as a notable demonstration of the efficacy of public-private partnerships in tackling urgent public health challenges [20].

Overall, the community environment improvement campaigns in Wad El Nimear village serve as a model for collaborative efforts to enhance environmental health and combat endemic diseases. Moreover, it engages the villagers in addressing their health concerns. By mobilising resources, expertise, and community participation, these campaigns have the potential to significantly improve the well-being of the villagers and serve as a blueprint for similar initiatives in other affected regions.

### Artificial limbs prosthesis mission for endemic villages

Delayed presentations of advanced disease and severe complications are common in cases of mycetoma, primarily due to several factors. These include the condition’s painless nature, patients’ limited health education, and their low socioeconomic status. Additionally, in remote and endemic regions, the scarcity of health services forces patients to travel long distances to specialised centres for diagnosis and treatment, resulting in financial strain and treatment delays. Consequently, late-stage disease often requires amputation or extensive surgical procedures, leading to significant disability and deformities. Amputation rates in mycetoma remain alarmingly high, ranging from 10% to 30%, with devastating socioeconomic and psychological repercussions for patients, their families, and communities [11,27,29]. These include social stigma, economic losses due to job displacement, educational disruption, and marital difficulties, and may lead to patients resorting to begging for livelihoods. Moreover, the utilisation of poorly designed, locally crafted prostheses exacerbates these challenges, as they are cumbersome, unsightly, and socially ostracising.

In response to these pressing challenges, the Mycetoma Research Center, in collaboration with the Ministry of Health, Sennar State, and EL Zaki Fund, initiated a national campaign to provide free artificial limb prostheses for mycetoma amputees in endemic villages across Sudan. A mobile prosthetic workshop, staffed with expert technicians and equipped with manufacturing equipment and physiotherapy resources, was deployed to the Mycetoma Satellite Centre in Wad Onsa village, Eastern Sennar Locality, Sennar State, Sudan.

The initiative received a warm reception from amputees, villagers, and local authorities alike. Over 3 weeks, the team successfully fitted 160 amputees, some of whom received bilateral prostheses while also providing training and physiotherapy services. The provision of prostheses significantly improved the quality of life for amputees and their families, enabling students to resume their education and individuals to engage in daily activities independently. Moreover, the campaign alleviated the financial burden on amputees and their families by eliminating the costs associated with prosthetic acquisition and travel to urban centres [20].

### Mycetoma vocational and Entrepreneurship training centre

The establishment of the Mycetoma Vocational and Entrepreneurship Training Centre, known as SAAI’D, marks a significant milestone in the efforts of the Mycetoma Research Center to address the multifaceted challenges faced by individuals affected by mycetoma and disabilities. Functioning as a cornerstone institution, SAAI’D embodies a commitment to vocational training, entrepreneurship development, and community engagement aimed at fostering empowerment and resilience among its participants [20].

At the heart of SAAI’D’s mission lies a vision to foster equality and eliminate the stigma surrounding mycetoma while empowering individuals through skill development and economic opportunities. Through a comprehensive array of vocational programmes and entrepreneurial initiatives, the center endeavours to equip its participants with the tools and knowledge necessary to navigate their paths towards self-reliance and success. From traditional handicrafts to modern entrepreneurship principles, SAAI’D offers a diverse range of training options tailored to meet the needs and interests of its diverse clientele.

Underpinning the operations of SAAI’D is a dedicated Board tasked with shaping policies and strategies, ensuring alignment with the center’s overarching goals. This governance structure ensures accountability and transparency in the center’s activities, guiding its efforts towards achieving tangible outcomes such as economic stability, poverty reduction, and enhanced public awareness of mycetoma.

Operating within eco-friendly facilities, SAAI’D embodies a commitment to sustainability and environmental stewardship, reflecting its holistic approach to community development. Through strategic partnerships and collaborations with businesses, NGOs, and governmental agencies, the center sustains its operations and expands its reach, ultimately making a meaningful impact in the lives of those it serves.

The establishment of SAAI’D was made possible through the generous support of the Embassy of Japan to Sudan, whose donations laid the foundation for its inception. Today, the center continues to thrive through ongoing contributions and support from various stakeholders, underscoring the collective commitment to advancing the well-being and empowerment of individuals affected by mycetoma and disabilities.

### Accessible treatment and support services

Given the poor socioeconomic and health literacy levels among affected individuals, it is imperative to establish accessible treatment and support services within their communities. This not only ensures proximity to care but also fosters community engagement. Central to this effort is advocacy for the establishment of specialised treatment centres, guaranteeing the affordability or provision of free medications and surgical procedures, and offering comprehensive psychosocial support. These multifaceted initiatives aim to alleviate financial constraints, mitigate emotional distress, and address the educational needs of both patients and their families.

However, the establishment and maintenance of such facilities pose significant financial burdens on local health authorities in endemic regions. Therefore, fostering collaboration among healthcare providers, community stakeholders, philanthropic organisations, and NGOs is crucial in realising these vital resources. Through joint efforts, including community engagement initiatives and donor support, sustainable solutions can be achieved. This collaborative endeavour exemplifies the principles of public-private partnership and underscores the corporate social responsibility of companies. By pooling resources and expertise, these partnerships can effectively address the complex challenges associated with providing healthcare services in underserved communities. The majority of the MRC’s initiatives aimed at delivering outstanding free medical, surgical, and socioeconomic services were carried out through such collaborative undertakings.

### Collaboration with local institutes

Collaborating with local institutions such as schools, religious organisations, and community centres offers a strategic approach to addressing mycetoma within communities. By utilising existing infrastructure and networks, stakeholders can broaden the scope and effectiveness of interventions aimed at prevention, education, and healthcare access. Schools serve as educational platforms for raising awareness among students, teachers, and parents, while religious organisations can disseminate messages through gatherings and community outreach. Community centres provide opportunities for health camps and workshops, facilitating access to healthcare services. By encouraging trust and sustainability through these collaborations, stakeholders can overcome barriers and mobilise communities for a long-term impact on mycetoma prevention and control efforts. The MRC utilised this collaboration effectively to execute its missions and objectives on mycetoma awareness and management.

### Community-based research

Research and innovation are crucial for advancing our understanding of mycetoma, encompassing epidemiology, risk factors, and diagnostics and treatment outcomes. Engaging communities in research efforts fosters collaboration, trust, and participation, enriching data collection and enhancing the relevance of findings. Studies focus on elucidating epidemiological patterns and assessing treatment effectiveness, with community involvement ensuring culturally sensitive approaches and addressing local needs. Community engagement spans research design, implementation, and knowledge exchange, empowering stakeholders and informing policy and practice for more equitable and effective mycetoma interventions. The MRC has conducted several field-based surveys and studies that have enriched the medical literature [10,21–25].

During the community engagement activities, the MRC embarked on a multifaceted research endeavour, which was only made possible through active community involvement. One critical aspect of this initiative was the implementation of an extensive epidemiological study in the Eastern Sennar locality, designed to elucidate the prevalence and burden of mycetoma. Through meticulous house-to-house surveys conducted in various study areas, demographic data was meticulously collected, while suspected cases were identified for further assessment and management [21–25].

Moreover, the scope of research extended beyond mere epidemiological investigations. Some immunological and genetic studies are continuing in locales such as Wad Onsa and Wad El Numir, aiming to unravel potential associations between specific immunogenetic and genetic factors and mycetoma incidence. These studies have delved into the intricate molecular underpinning of the disease, shedding light on its complex aetiology.

In addition to human-focused research, comprehensive examinations of mycetoma geographic distribution were undertaken. These investigations honed in on environmental determinants, meticulously mapping out the disease’s prevalence in relation to factors such as soil composition, vegetation, water sources, and proximity to livestock. By analysing these environmental factors, researchers sought to uncover potential hotspots for mycetoma transmission and identify areas ripe for targeted intervention strategies [31].

In essence, these concerted efforts underscore a commitment to not only deepening scientific understanding but also to empowering local communities in combating mycetoma. By integrating community participation into the research process, the MRC has fostered a collaborative approach that not only generated valuable data but also empowered communities to take an active role in addressing the health challenges they face. Through these endeavours, a more nuanced understanding of mycetoma and its environmental determinants emerged, paving the way for more effective prevention and management strategies in Sennar State and beyond.

In conclusion, the extensive experience of The Mycetoma Research Center in community engagement over 3 decades has provided valuable insights and lessons. Firstly, establishing clear objectives and fostering close collaboration with local health, administrative, and political authorities is paramount for conducting effective and meaningful community engagement initiatives. Additionally, the active involvement and support of the community, including its leaders, volunteers, and the entire population, play fundamental roles in shaping the success of engagement efforts. Empowering the community to participate in determining its health and socioeconomic priorities is essential. Moreover, adhering to high-quality ethical principles such as respecting individual autonomy, preventing harm, and ensuring transparency is essential for conducting humane community engagement activities. Strategic collaboration with diverse stakeholders is crucial for achieving engagement objectives, and leveraging public-private partnerships can lead to significant accomplishments. Furthermore, it’s important to engage with both high-achieving individuals and those who may require additional support, fostering a collaborative environment that encourages growth and achievement for all involved parties.

**Learning Points**

- Community engagement initiatives are required to encourage villages to take part in their healthcare responsibilities.
- Holistic mycetoma management is the key to sustainable, good healthcare.
- Mycetoma stockholders’ contributions are mandatory for good treatment outcomes.
- The challenges posed by mycetoma are significant, particularly in resource-limited settings; hence, community engagement and support are mandatory.

**Key papers in the field**

- Bakhiet SM, Fahal AH, Musa AM, Mohamed ESW, Omer RF, Ahmed ES, El Nour M, Mustafa ERM, Sheikh A Rahman ME, Suliman SH, El Mamoun MAG, El Amin HM. A holistic approach to the mycetoma management. PLoS Negl Trop Dis. 2018 May 10;12(5):e0006391. doi: 10.1371/journal.pntd.0006391. PMID: 29746460; PMCID: PMC5944909.
- Mohamed ESW, Bakhiet SM, El Nour M, Suliman SH, El Amin HM, Fahal AH. Surgery in mycetoma-endemic villages: unique experience. Trans R Soc Trop Med Hyg. 2021 Apr 14;115(4):320–323. doi: 10.1093/trstmh/traa194. PMID: 33515452.
- Fahal A, Mahgoub el S, El Hassan AM, Abdel-Rahman ME, Alshambaty Y, Hashim A, Hago A, Zijlstra EE. A new model for management of mycetoma in the Sudan. PLoS Negl Trop Dis. 2014 Oct 30;8(10):e3271. doi: 10.1371/journal.pntd.0003271. PMID: 25356640; PMCID: PMC4214669.
- Abbas M, Scolding PS, Yosif AA, El Rahman RF, El-Amin MO, Elbashir MK, Groce N, Fahal AH. The disabling consequences of Mycetoma. PLoS Negl Trop Dis. 2018 Dec 10;12(12):e0007019. doi: 10.1371/journal.pntd.0007019. PMID: 30532253; PMCID: PMC6312340.
- Gabani MH, Ahmed AA, Hassan AA, Abdalla MA, Mustafa SA, Alobaid TA, Khatir AA, Mohammed RM, Awad NI, Abdellateef TA, Hassan A, Ahmed ES, Ali MZ, Fahal AH. The nutritional status of mycetoma affected patients seen at the Mycetoma Research Center, Sudan. PLoS Negl Trop Dis. 2024 Jan 2;18(1):e0011726. doi: 10.1371/journal.pntd.0011726. PMID: 38166142; PMCID: PMC10786388.

### The Advantages

- Community engagement initiatives will integrate efforts to better treatment outcomes.
- Community engagement will reduce healthcare cost
